# Editorial Note: DNA persistence and relapses questions on the treatment strategies of *Enterococcus* infections of prosthetic valves

**DOI:** 10.1371/journal.pone.0346848

**Published:** 2026-04-09

**Authors:** The PLOS One Editors

The *PLOS One* Editors issue this notice to update the previously published Expression of Concern on this article [1,2].

Following the publication of the article and Expression of Concern [1,2], PLOS investigated concerns pertaining to the reported ethical approval and the article’s adherence to *PLOS One’s* research ethics and reporting requirements.

Specifically, the research ethics concerns included that the study involved human participants but did not receive ethics approval from a Comité de Protection des Personnes, and that the ethics approval number #07–015 was also reported in multiple other publications [3–5], despite there appearing to be differences in the periods during which data were collected and types of consent obtained from the participants described in these studies.

A representative of the Aix-Marseille Université Ethics Committee stated that the institutional investigation into the ethics concerns concluded this article meets ethical standards. They commented that the study described in [1] did not involve invasive or risky sampling as the study is based solely on the analysis of heart valves collected as part of routine patient care, and that the study did not require ethics approval from a Comité de Protection des Personnes according to French law. The representative provided the ethics approval document #07–015 for editorial review.

Ethics approval document #07–015 was issued on 19 February 2007 by the Comité d’Ethique de l’IRF48 for a study titled “*Diagnosis of blood culture-negative endocarditis: what is left when you have searched all microorganisms*”. It allows for the use of blood samples and heart valves originating from the bacteriology laboratory of Timone Hospital, Marseille, France.

PLOS reviewed the documentation provided by the institution and concluded that the documents did not fully resolve the concerns. Specifically,

- The article does not report when the samples used in this study were collected. This information is needed to evaluate the article’s compliance with the PLOS Human Subjects Research policy.
- The ethics approval document was subject to conditions, but the details of these conditions were not legible to the journal’s editorial team, and the institute did not reply to requests for clarification of this text.
- One of the ethics committee members listed on the ethics approval document is a co-author of this article [1].
- PLOS identified additional potential competing interests between the committee that granted the ethics approval and one or more of the article’s authors.

In light of the unresolved issues, the Expression of Concern stands.
